# Exclusive breastfeeding among women taking HAART for PMTCT of HIV-1 in the Kisumu Breastfeeding Study

**DOI:** 10.1186/1471-2431-14-280

**Published:** 2014-11-07

**Authors:** John O Okanda, Craig B Borkowf, Sonali Girde, Timothy K Thomas, Shirley Lee Lecher

**Affiliations:** Kenya Medical Research Institute/U.S. Centers for Disease Control and Prevention (KEMRI/CDC), Research and Public Health Collaboration, P.O. Box 1578, 40100 Kisumu, Kenya; Division of HIV/AIDS Prevention, U.S. Centers for Disease Control and Prevention, Atlanta, GA USA; ICF International, Atlanta, GA USA; U.S. Centers for Disease Control and Prevention, Kisumu, Kenya

## Abstract

**Background:**

One of the most effective ways to promote the prevention of mother-to-child transmission (PMTCT) of HIV-1 in resource-limited settings is to encourage HIV-positive mothers to practice exclusive breastfeeding (EBF) for the first 6 months post-partum while they receive antiretroviral therapy (ARV). Although EBF reduces mortality in this context, its practice has been low. We studied the rate of adherence to EBF and assessed associated maternal and infant characteristics using data from a phase II PMTCT clinical trial conducted in Western Kenya which included a counseling intervention to encourage EBF by all participants.

**Methods:**

We analyzed data from the Kisumu Breastfeeding Study (KiBS), conducted between July 2003 and February 2009. This study enrolled a total of 522 HIV-1 infected pregnant women. Data on breastfeeding were available for 480 mother-infant pairs. Infant feeding and general nutrition counseling began at 35 weeks gestation and continued throughout the 6 month post-partum intervention period, following World Health Organization (WHO) infant feeding guidelines. Data on infant feeding were collected during routine clinic visits and home visits using food frequency questionnaires and dietary recall methods. Participants were instructed to exclusively breastfeed until initiation of weaning at 5.5 months post-partum. We used Kaplan-Meier methods to estimate the rates of EBF at 5.25 months post-partum, stratified by maternal and infant characteristics measured at enrollment, delivery, and 2 weeks post-partum.

**Results:**

The estimated EBF rate at 5.25 months post-partum was 80.4%. Only 3% of women introduced other foods (most commonly water with or without glucose, cow’s milk, formula, and fruit) by 2 months; this percentage increased to 5% of women by 4 months. Women who had ≥3 previous births (p < 0.01) and who were not living with the infant’s father (p = 0.04) were more likely to exclusively breastfeed. Mixed feeding was more common for male infants than for female infants (p = 0.04).

**Conclusion:**

Exclusive breastfeeding was common in this clinical trial, which emphasized EBF as a best practice until infants reached 5.5 months of age. Counseling initiated prior to delivery and continued during the post-partum period provided a consistent message reinforcing the benefits of EBF. The findings from this study suggest high adherence to EBF in resource limited settings can be achieved by a comprehensive counseling intervention that encourages EBF.

## Background

For the prevention of mother-to-child transmission (PMTCT) of HIV-1 in resource limited settings, the World Health Organization (WHO) recommends that HIV-infected mothers receive antiretroviral therapy (ARV) and practice exclusive breastfeeding (EBF) for the first 6 months post-partum followed by complementary feeding unless environmental and social circumstances are safe for and supportive of replacement feeding [1]. In addition to the significant gains in reducing HIV transmission through PMTCT prophylaxis during pregnancy, studies have shown an association between EBF and decreased HIV transmission in the first 6 months of infant life compared to mixed feeding [2–8]. Despite WHO recommendations promoting EBF practices, adherence to EBF is often low [7, 9–12]. EBF remains a challenge in sub-Saharan Africa, as it culturally more acceptable to provide mixed feeding with supplemental fluids or other foods traditional for infants [11, 12]. Globally, less than 40% of infants under 6 months of age are exclusively breastfed [13]. In sub-Saharan Africa, pre-lacteal feeding [10, 14], mixed feeding as early as 2 weeks after delivery, and breastfeeding beyond 2 years are common [15]. Multiple studies have highlighted various predictors of breastfeeding practices, such as societal norms [16], cultural beliefs [14, 17, 18], age [19–23], socioeconomic status [11, 14, 24], medical problems [16, 21, 25], psychosocial factors [26], and family influence [12, 17, 27–32]. While some studies have focused on the challenges of EBF, there is limited data from clinical trials in this region on the successes of EBF.

The focus of this analysis was to estimate the proportion of mothers who adhered to the current recommendations to exclusively breastfeed for 5.25 months in the context of a PMTCT clinical trial with access to appropriate ARV therapy. Factors previously known to be associated with EBF, such as age [19–23] and socioeconomic status [11, 14, 24], among others, were examined to determine whether they increased or decreased adherence to EBF during the first 6 months post-partum.

## Methods

This analysis uses data obtained from participants enrolled in the Kisumu Breastfeeding Study (KiBS), an open label phase IIb PMTCT clinical trial conducted between July 2003 and February 2009 (ClinicalTrials.gov NCT00146380). A detailed description of the study design and methods has previously been published [33]. In brief, HIV-infected pregnant women attending PMTCT programs at the antenatal clinics of the New Nyanza Provincial General Hospital and the Kisumu District Hospital, which serve lower income populations of Kisumu, were counseled on the risks and benefits of various infant feeding options. After receiving infant feeding counseling, women who indicated intent to breastfeed and met other study inclusion criteria were enrolled at 34 weeks gestation. All study participants were ARV naïve and received triple-combination ARV therapy consisting of zidovudine/lamivudine coformulated as combivir (GlaxoSmithKline) with either nevirapine (Viramune, Boehringer Ingelheim) or nelfinavir (Viracept, Hoffman-La Roche Ltd.). Participants received ARV therapy from 34 weeks gestation through 6 months post-partum. Plasma viral load was quantified using the Amplicor HIV-1 RNA Monitor Test v1.5 (Roche Diagnostics). CD4 lymphocytes were analyzed on a FACSCalibur flow cytometer (Becton Dickinson).

In accordance with WHO guidelines, women were counseled to exclusively breastfeed their infants for 5.5 months and then to wean them promptly over a 2-week period, with complete cessation of breastfeeding by 6 months post-partum. ARVs were discontinued at 6 months post-partum unless the participants met WHO guidelines for their own treatment at the time of the study. EBF was defined as feeding with only breast milk. Medicines and herbs for medicinal purposes were allowed. Mixed feeding was defined as the ingestion of any liquid or food in addition to breast milk. The use of locally available liquids and foods (e.g., porridge, soups, fruit juices, and cow’s milk) was encouraged for weaning and replacement feeding.

The study protocol was approved by the U.S. Centers for Disease Control and Prevention (CDC) Institutional Review Board in Atlanta and the Kenya Medical Research Institute (KEMRI) Ethics Review Committee. All participants provided written consent before enrolling in the study.

### Data collection and analysis

Infant feeding and general nutrition counseling began after enrollment at 34 weeks gestation. Participants were seen weekly at the clinic for clinical evaluation and received counseling on: maternal nutrition, preparation for lactation, importance of EBF, good breastfeeding techniques, breast health, common breastfeeding problems and strategies for reducing breast milk transmission of HIV in a breastfeeding infant. Home visits also occurred weekly after enrollment at 34 weeks gestation by social workers with certificates in counseling and social work to assess maternal progress since starting the study drugs and to reinforce the benefits of EBF, in preparation for lactation. The time spent for home visits averaged 40 to 60 minutes. After delivery, each mother-infant pair was seen in the clinic for follow-up at 2, 6, 10, and 14 weeks post-partum by a clinician and a nutritionist. During clinic visits participants received counseling to reinforce EBF and an assessment of breast health. Data was collected at each of the visits. At 5 months the mother-infant pair was seen at the clinic by the nutritionist, who discussed with them making the transition from EBF to replacement feeding at 5.5 months post-partum. Mothers and infants continued to be followed at 6, 9, 12, 15, 18, and 24 months post-partum. Three clinic visits were later added at 5, 7, and 8 months post-partum to monitor infant growth around the weaning period. During study visits adherence to medications and infant feeding recommendations were assessed by study coordinators. Infant feeding counseling was carried out using WHO 2006 guidelines for HIV and infant feeding. In addition, home visits were conducted with monitoring of infant feeding between the clinic visits at 1, 4, 8, 12, and 16 weeks and at 6, 7, 8, 10, 11, 14, 17, 20, and 22 months post-partum. During home visits the interviewer directly observed the home environment and interviewed other household members caring for the infant to assess whether the infant was being fed other foods or still breastfeeding. During each interview conducted at clinic and home visits, data was collected through face-to-face interviews with participants and via observation on infant feeding practices, including: breastfeeding, breast health problems, introduction of complementary liquids or foods, and cessation of breastfeeding. Data from both interviews and direct observations were recorded on case report forms and were considered equally in determining when mixed feeding first occurred. Any report of liquids or foods other than breast milk was considered to be evidence of mixed feeding.

The time of the first mixed feed was calculated based on data from multiple sources: the current breastfeeding question in the clinic visit form, the current breastfeeding question and evidence of current mixed feeding (e.g., baby seen eating other foods, food on baby’s clothes) in the home visit form, the breastfeeding cessation date form, and a mixed feeding form. This last form for mixed feeding lists the liquids and foods other than breast milk, (i.e., water, glucose water, tea, juice, formula, fresh milk, porridge, and fruits) given to the infant over 3 days prior to the current visit, if the participant reported either having difficulty maintaining EBF or having practiced mixed feeding. Case report forms for recording data were processed by an automated teleform system.

Statistical analyses were performed on data obtained on 480 mothers and their live-born infants for whom any breastfeeding data were available. The cumulative counts and percentages of various liquids and foods were computed by time after delivery for visits scheduled at 2, 6, 10, and 14 weeks and 5 and 6 months post-partum (corresponding to 14, 42, 70, 98, 152, and 183 days post-partum, respectively). Measurements that occurred within 2 weeks (14 days) after each scheduled visit were included with that scheduled visit. Summary statistics were computed for various maternal and infant socio-demographic and clinical characteristics. Kaplan-Meier methods were used to calculate the rates of EBF at 5.25 months (160 days) post-partum, stratified by maternal and infant characteristics measured at enrollment, delivery, and 2 weeks post-partum. The event of interest was time of first mixed feeding, and observations were censored for loss to follow-up and withdrawal from the study. In addition, 95% confidence intervals were computed on the complementary log-log scale using the Greenwood variance estimator. Cochran’s Q test was used to test for heterogeneity among the strata in the estimated EBF rates at 5.25 months post-partum. A 5% significance level was chosen. All computations were performed using SAS software, version 9.3 (SAS Institute, Inc., Cary, NC).

## Results

The study enrolled 522 HIV-1 infected pregnant women, of whom 491 delivered 502 live infants. Data on breastfeeding were available for 480 mother-infant pairs.

Maternal baseline socio-demographic and clinical characteristics are listed in Table 1. The largest proportion of women (43%) were between 20–24 years of age, 74% were married, 72% had completed primary education, 35% were employed outside of the home, and 84% were at WHO clinical disease stage 1.

**Table 1:** **Rates of exclusive breastfeeding at 5.25 months post-partum, stratified by maternal characteristics measured at enrollment, for 480 mothers with breastfeeding data**

			EBF at 5.25 mo. post-partum*		
Variable (n = 480)	Category	Number (%) or median (Range)	KM rate (%)	95% CI (%)	p-value**
Overall	NA	480 (100%)	80.4	(76.5, 83.7)	NA
Ethnic group	Luo	409 (85%)	80.0	(75.7, 83.6)	0.80
	Luhya	50 (10%)	83.7	(70.1, 91.5)	
	Other	21 (4%)	81.0	(56.9, 92.4)	
Age (years)	15 – 19	64 (13%)	75.6	(62.7, 84.5)	0.50
	20 – 24	208 (43%)	78.8	(72.6, 83.9)	
	25 – 29	132 (28%)	83.8	(76.2, 89.1)	
	30 – 43	76 (16%)	82.5	(71.8, 89.5)	
Median age (years)		23 (15 – 43)			
Primigravid	Yes	113 (24%)	79.9	(71.1, 86.3)	0.89
	No	367 (76%)	80.5	(76.0, 84.2)	
Median parity, among multigravid (n = 392)		2 (0 – 8)			
If not primigravid, number of live births (n = 367)	0 – 2	278 (76%)	77.4	(72.0, 82.0)	<0.01
	3 – 8	89 (24%)	89.8	(81.4, 94.6)	
Marital status	Single	62 (13%)	84.7	(72.6, 91.7)	0.07
	Married	357 (74%)	78.5	(73.8, 82.5)	
	Separated/divorced	25 (5%)	79.2	(57.0, 90.8)	
	Widowed	36 (8%)	91.7	(76.3, 97.2)	
Living with child’s father (n = 479)	Yes	346 (72%)	78.1	(73.3, 82.2)	0.04
	No	133 (28%)	86.0	(78.7, 91.0)	
Number of adults in household (n = 479)	1	16 (3%)	80.0	(50.0, 93.1)	0.43
	2	129 (27%)	75.9	(67.4, 82.5)	
	3	126 (26%)	78.5	(70.2, 84.7)	
	4 – 7	186 (39%)	84.0	(77.8, 88.6)	
	8 – 14	22 (5%)	85.7	(62.0, 95.2)	
Median people in household (n = 470)		3 (1 – 14)			
Completed primary education (8 years)	Yes	345 (72%)	81.0	(76.4, 84.8)	0.60
	No	135 (28%)	78.8	(70.8, 84.9)	
Highest level of education attended (n = 467)	Primary	286 (61%)	78.6	(73.3, 83.0)	0.14
	Secondary	162 (35%)	84.7	(78.1, 89.5)	
	Tertiary	19 (4%)	68.4	(42.8, 84.4)	
Mother employed outside of home	Yes	166 (35%)	79.3	(72.1, 84.8)	0.68
	No	314 (65%)	80.9	(76.1, 84.9)	
If employed, occupation of mother (n = 166)	Professional/technical/managerial	24 (14%)	73.4	(50.1, 87.1)	0.49
	All others	142 (86%)	80.3	(72.6, 86.0)	
Level of income (KSh^§^/month) (n = 478)	<2,000	61 (13%)	77.8	(64.8, 86.5)	0.90
	2,000 – 4,999	98 (21%)	80.6	(71.3, 87.2)	
	5,000 – 9,999	58 (12%)	76.6	(63.0, 85.7)	
	≥10,000	36 (8%)	79.2	(61.3, 89.5)	
	Unknown	225 (47%)	81.9	(76.2, 86.4)	
Viral load (copies/ml) (n = 478)	Undetectable (<400)	27 (6%)	74.1	(53.2, 86.7)	0.28
	400 – 9,999	110 (23%)	84.1	(75.7, 89.8)	
	10,000 – 49,999	138 (29%)	75.5	(67.3, 81.9)	
	≥50,000	203 (42%)	82.3	(76.3, 87.0)	
CD4 count (cells/mm3)	0 – 199	70 (15%)	82.7	(71.5, 89.8)	0.50
	200 – 349	119 (25%)	82.8	(74.7, 88.6)	
	350 – 499	135 (28%)	81.8	(74.1, 87.4)	
	≥500	156 (33%)	76.2	(68.6, 82.2)	
WHO clinical HIV disease stage	Stage 1	403 (84%)	81.5	(77.3, 85.0)	0.38
	Stage 2	44 (9%)	71.7	(55.5, 82.8)	
	Stage 3 or 4	33 (7%)	78.5	(60.0, 89.1)	
Body mass index (BMI) at enrollment (n = 479)	15.00 – 18.49	5 (1%)	100.0	(47.8, 100.0)	0.72
	18.50 – 24.99	326 (68%)	79.0	(74.1, 83.1)	
	25.00 – 29.99	121 (25%)	82.9	(74.8, 88.6)	
	30.00 – 45.00	27 (6%)	81.5	(61.1, 91.8)	
Initial triple-ARV regimen	ZDV/3TC plus NVP	283 (59%)	79.5	(74.2, 83.8)	0.54
	ZDV/3TC plus NFV	197 (41%)	81.7	(75.5, 86.5)	
Disclosed HIV status to husband, child’s father, or partner (n = 302)	Yes	216 (72%)	83.1	(77.3, 87.5)	0.58
	No	86 (28%)	85.6	(76.1, 91.6)	

*The Kaplan-Meier (KM) method was used to estimate the rates of EBF at 5.25 months (160 days) post-partum, stratified by maternal characteristics measured at enrollment. The 95% confidence intervals (CIs) were calculated using the Greenwood variance estimator on the log-log scale.

**The p-value is for Cochran’s Q test of homogeneity at 5.25 months.

^§^The exchange rate is approximately 85 Kenyan Shillings (KSh) to 1 US dollar (rate varies).

The majority of women in the study adhered to the suggested guidelines to exclusively breastfeed up to 5.5 months post-partum, as indicated by the Kaplan-Meier analysis in Table 1. At 5.25 months post-partum, 80.4% of those in the study were still exclusively breastfeeding. Two of the maternal characteristics measured at enrollment (Table 1) were associated with EBF. Among multigravid women, those who had ≥3 children were more likely to exclusively breastfeed than those who had 0–2 children (89.8% vs. 77.4%, p < 0.01). Also, women who were not living with the child’s father were more likely to exclusively breastfeed than women who were living with the child’s father (86.0% vs. 78.1%, p = 0.04). When we examined maternal and infant characteristics measured at delivery and 2 weeks post-partum (Table 2), female infants were more likely to be exclusively breastfed compared to male infants (84.5% vs. 76.9%, p = 0.04). None of the other variables were significantly associated with EBF at the 5% significance level. When mixed feeding was practiced prior to the suggested weaning period, the most commonly given liquids and foods were milk, water with or without glucose, fruit, and porridge (Table 3). Only 3% and 5% of women introduced fluids other than breast milk by 2 and 4 months post-partum, respectively. Only 4 women gave their infants formula prior to 6 months post-partum, and the percentage remained small even during the period of suggested weaning.

**Table 2:** **Rates of exclusive breastfeeding at 5.25 months post-partum, stratified by maternal and infant (first-born, if multiple births) characteristics measured at delivery and two weeks post-partum, for 480 mothers with breastfeeding data**

			EBF at 5.25 mo. post-partum*		
Variable (n = 480)	Category	Number (%)	KM rate (%)	95% CI (%)	p-value**
WHO clinical HIV disease stage at 2 weeks after delivery (n = 461)	Stage 1	410 (89%)	81.8	(77.7, 85.3)	0.66
	Stage 2	24 (5%)	74.1	(51.1, 87.4)	
	Stage 3 or 4	26 (6%)	84.3	(63.3, 93.8)	
Body mass index (BMI) at 2 weeks post-partum (n = 461)	15.00 – 18.49	38 (8%)	75.9	(58.8, 86.7)	0.79
	18.50 – 24.99	365 (79%)	81.2	(76.8, 84.9)	
	25.00 – 29.99	47 (10%)	84.9	(70.9, 92.5)	
	30.00 – 45.00	11 (2%)	81.8	(44.7, 95.1)	
Infant birth weight (g) <2500 (n = 476)	Yes	37 (8%)	80.6	(63.5, 90.3)	0.98
	No	439 (92%)	80.5	(76.4, 83.9)	
Infant sex	Male	260 (54%)	76.9	(71.3, 81.6)	0.04
	Female	220 (46%)	84.5	(78.9, 88.7)	
Location of delivery	Hospital	377 (79%)	80.2	(75.8, 83.9)	0.98
	Other facility	21 (4%)	84.2	(58.7, 94.6)	
	Home	45 (9%)	79.5	(64.2, 88.8)	
	Other’s home	29 (6%)	82.1	(62.3, 92.1)	
	Other/unknown	8 (2%)	75.0	(31.5, 93.1)	
Delivery method	Spontaneous vertex	369 (77%)	80.6	(76.1, 84.3)	0.26
	Breech	6 (1%)	66.7	(19.5, 90.4)	
	Cesarean	34 (7%)	69.2	(50.2, 82.1)	
	Unknown	71 (15%)	85.7	(75.1, 92.1)	
Time to breastfeeding initiation (n = 460)	<1 hour	283 (62%)	79.5	(74.2, 83.8)	0.07
	1 – 6 hours	119 (26%)	77.5	(68.8, 84.1)	
	7 – 48 hours	58 (13%)	89.4	(77.9, 95.1)	
Prelacteal feed (n = 472)	Yes	23 (5%)	78.0	(55.0, 90.2)	0.81
	No	449 (95%)	80.1	(76.1, 83.6)	

*The Kaplan-Meier (KM) method was used to estimate the rates of EBF at 5.25 months (160 days) post-partum, stratified by maternal and infant (first-born, if multiple births) characteristics measured at delivery and two weeks post-partum. The 95% confidence intervals (CIs) were calculated using the Greenwood variance estimator on the log-log scale.

**The p-value is for Cochran’s Q test of homogeneity at 5.25 months.

**Table 3:** **Cumulative numbers and percentages of infants introduced to various liquids and foods by time after delivery, for 480 mothers and infants with any breastfeeding data**

	Time after delivery*					
	2 weeks	6 weeks	10 weeks	14 weeks	5 months	6 months
Liquid or food	n (%)	n (%)	n (%)	n (%)	n (%)	n (%)
Formula	-	4 (1)	4 (1)	4 (1)	4 (1)	13 (3)
Water	-	3 (1)	5 (1)	6 (1)	21 (4)	283 (59)
Glucose water	1 (<1)	3 (1)	3 (1)	4 (1)	10 (2)	76 (16)
Milk, cow or goat	-	3 (1)	8 (2)	10 (2)	29 (6)	316 (66)
Juice	-	-	-	2 (<1)	9 (2)	134 (28)
Porridge	-	-	-	4 (1)	24 (5)	352 (73)
Fruit	-	3 (1)	5 (1)	5 (1)	18 (4)	205 (43)
Tea	-	-	-	-	-	81 (17)
Combined**	1 (<1)	12 (3)	20 (4)	24 (5)	53 (11)	408 (85)

*Visits were scheduled at 2, 6, 10, and 14 weeks and 5 and 6 months (corresponding to 14, 42, 70, 98, 152, and 183 days, respectively). Measurements that occurred within two weeks (14 days) after each scheduled visit were included with that scheduled visit.

**The combined category refers to the numbers and percentages of infants introduced to any of the eight listed liquids and foods by certain times after delivery.

## Discussion

Our findings of 80.4% overall adherence to EBF at 5.25 months post-partum in this study is much higher than expected, given global estimates of 39% and Kenya national estimates of 32% [34]. A likely explanation for the higher EBF adherence in this study was the comprehensive support provided to participants in this PMTCT clinical trial. After enrollment, participants were engaged with clinic personnel who provided frequent reinforcement to adhere to EBF for 5.5 months until weaning. Likewise, a KiBS sub-study that focused on a safe water intervention also reported high adherence (80% to 90%) to the recommended chlorine practices and a low frequency of clinic visits for diarrheal cases during the EBF period [35]. In contrast to our findings of high adherence to recommendations for EBF until 5.5 months post-partum, a recent study to examine feeding practices among HIV-infected and uninfected mothers in Kisumu, Kenya, where this clinical trial was conducted, found only 14% of HIV-infected women practiced EBF for the first 6 months post-partum [24]. We assert that high adherence to EBF may be a positive result of providing counseling and routine follow-up in this clinical trial setting. Providing regular and consistent messaging to mothers, as was done in this PMTCT clinical trial, can facilitate compliance with WHO breastfeeding recommendations and best practices in resource limited settings.

In this study we found that the EBF rate was higher among multigravid women who had ≥3 children prior to enrollment than among those with 0–2 children. One possible explanation for this finding is that women may find it easier to exclusively breastfeed having had previous experience with breastfeeding. Also, EBF is less expensive than mixed feeding, and cost may be a concern in larger families. Likewise, the EBF rate was higher among women not living with the child’s father than among women living with the child’s father. Women not living with the child’s father may find more time to spend alone with their infant, making EBF more convenient. In addition, the mixed feeding rate was higher among male infants than among female infants. This observation may reflect the cultural context that places higher regard on male infants in this region of Kenya and therefore seeks to give them the best nutrition and care. Thus, there may be a greater likelihood to start mixed feeding male infants earlier because supplementing breast milk with nutritious foods is thought to promote growth and development [14].

There are several limitations to our analysis. The high rate of EBF might be related to recruitment into the clinical trial of women who planned to breastfeed. However, the high rate of EBF reflects not only the fact that that these women breastfed their infants, but also the fact that they refrained from mixed feeding, in contrast to the most common practice in Kenya, as in the rest of sub-Saharan Africa. Furthermore, there were frequent clinic and home visits with continuous counseling and support for EBF, which likely impacted infant feeding practices. Social desirability bias may have been introduced through individuals answering questions that they exclusively breastfed since this is what was expected of them and considered most appropriate rather than what was actually done. Nevertheless, attempts were made to observe infant behavior and feeding practices in the home by social workers during home visits. Feeding habits were also discussed with family members during home visits.

Clinical trials and other interventions that provide education and reinforcement, such as peer counselors, can have a positive impact on improving the knowledge and attitudes about EBF [30]. Progress to increase the number of women who exclusively breastfeed has been reported on a large scale in Africa and Latin America through training, effective behavior change communication and the development of key partnerships which influenced communities [15, 36]. It may be challenging to translate the experience from this clinical trial to busy clinic settings in resource constrained areas with limited staff. Use of community volunteers to emphasize the importance of EBF could prove valuable in these settings. Use of peer counselors in the PROMISE EBF study demonstrated a significant increase in EBF prevalence for women who were randomized to a peer counseling intervention which involved one antenatal and four post-delivery breastfeeding peer counseling sessions, compared to a control group. The EBF prevalence at 12 weeks post-delivery in the intervention and control clusters was 79% vs. 35% in Burkina Faso, 82% vs. 44% in Uganda, and 10% vs. 6% in South Africa [37]. The interpretation from this study was that low intensity individual peer counseling can be used to effectively increase EBF prevalence in sub-Saharan African settings. These results and ours suggest that education and counseling from health care providers and community workers in resource-limited settings can improve adherence to the current breastfeeding guidelines.

## Conclusion

Health care and community workers can have a pivotal role in providing accurate information on the benefits of EBF, leading to optimal feeding practices and decreased infant morbidity and mortality. In resource-limited settings, strategies to promote EBF and to discourage early mixed feeding should be incorporated routinely within maternal child health programs.
